# Vitamin D supplementation is effective for olanzapine-induced dyslipidemia

**DOI:** 10.3389/fphar.2023.1135516

**Published:** 2023-02-21

**Authors:** Zijian Zhou, Takuya Nagashima, Chihiro Toda, Mone Kobayashi, Takahide Suzuki, Kazuki Nagayasu, Hisashi Shirakawa, Satoshi Asai, Shuji Kaneko

**Affiliations:** ^1^ Department of Molecular Pharmacology, Graduate School of Pharmaceutical Sciences, Kyoto University, Kyoto, Japan; ^2^ Division of Pharmacology, Department of Biomedical Sciences, Nihon University School of Medicine, Tokyo, Japan

**Keywords:** olanzapine, vitamin D, C2C12 cells, dyslipidemia, clinical big data, low-density lipoprotein, high-density lipoprotein

## Abstract

Olanzapine is an atypical antipsychotic drug that is clinically applied in patients with schizophrenia. It increases the risk of dyslipidemia, a disturbance of lipid metabolic homeostasis, usually characterized by increased low-density lipoprotein (LDL) cholesterol and triglycerides, and accompanied by decreased high-density lipoprotein (HDL) in the serum. In this study, analyzing the FDA Adverse Event Reporting System, JMDC insurance claims, and electronic medical records from Nihon University School of Medicine revealed that a co-treated drug, vitamin D, can reduce the incidence of olanzapine-induced dyslipidemia. In the following experimental validations of this hypothesis, short-term oral olanzapine administration in mice caused a simultaneous increase and decrease in the levels of LDL and HDL cholesterol, respectively, while the triglyceride level remained unaffected. Cholecalciferol supplementation attenuated these deteriorations in blood lipid profiles. RNA-seq analysis was conducted on three cell types that are closely related to maintaining cholesterol metabolic balance (hepatocytes, adipocytes, and C2C12) to verify the direct effects of olanzapine and the functional metabolites of cholecalciferol (calcifediol and calcitriol). Consequently, the expression of cholesterol-biosynthesis-related genes was reduced in calcifediol- and calcitriol-treated C2C12 cells, which was likely to be mediated by activating the vitamin D receptor that subsequently inhibited the cholesterol biosynthesis process *via* insulin-induced gene 2 regulation. This clinical big-data-based drug repurposing approach is effective in finding a novel treatment with high clinical predictability and a well-defined molecular mechanism.

## Introduction

Schizophrenia is a group of chronic psychiatric disorders characterized by hallucinations, delusions, reduced motivation, and blunt affect (Saha et al., 2005). Atypical antipsychotics are currently used as first-line drugs in patients with schizophrenia because they potently improve positive symptoms without causing extrapyramidal symptoms (Campbell et al., 1999). Among these, olanzapine is one of the most widely used drugs (Kochi et al., 2017). However, olanzapine causes dyslipidemia as a significant adverse effect, characterized by an increase in total blood cholesterol and triglyceride levels (Lieberman et al., 2005). A recent meta-analysis further revealed a relationship between olanzapine treatment and an increase in low-density lipoprotein (LDL) cholesterol in the blood (Li et al., 2020). Although olanzapine has excellent cardiac safety (Kennedy et al., 2001), the obesity induced by olanzapine treatment has been proven to be a cardiovascular risk factor (Wang et al., 2014; Correll et al., 2015), thereby making its treatment a significant risk factor that contributes to excess premature mortality in patients with schizophrenia (Brown et al., 2000). At present, preventing this adverse effect is impossible due to the incomplete understanding of the underlying mechanisms of olanzapine-induced dyslipidemia (Yan et al., 2013). Thus, there exists a clinical demand for novel target-screening approaches.

Drug repurposing from market-accessible drugs is a classic approach for refining the potential therapeutic applications. Repurposing studies have shown that topiramate, a newer anticonvulsant, was able to attenuate the obesity-inducing effect of olanzapine (Narula et al., 2010), and diabetes-treating drugs, such as metformin (Chen et al., 2008) and liraglutide (Larsen et al., 2017) have relieved the excessive body weight gain due to olanzapine treatment. However, a recently published meta-analysis review on the clinical trials focusing on blood lipid levels concluded that the available lipid-lowering agents are not effective in treating patients who are prescribed with atypical antipsychotics, including olanzapine (Kanagasundaram et al., 2021). Therefore, current practices need to be optimized, considering that existing drug repurposing approaches are inefficient in refining interventions with high clinical efficiency for treating olanzapine-induced dyslipidemia.

In this study, an updated approach, “reverse translational drug repurposing” (Kaneko and Nagashima, 2020), was adopted, making it viable to conduct a retrospective analysis with clinical big data and screen for hypothetical drug-drug pairs that potently ameliorate olanzapine-induced dyslipidemia in patients. We have previously utilized the self-reports of adverse events extracted from the FDA Adverse Events Reporting System (FAERS) to study the underlying mechanisms of hyperglycemia induced by an atypical antipsychotic, quetiapine, and identified the agent with treatment potential (Nagashima et al., 2016). Furthermore, to improve the drawbacks of FAERS (lacking time stamps and the population size of patients who were prescribed the specified drugs), another source of real-world clinical big data, insurance claims from JMDC Inc., was utilized in our recently published studies (Nagaoka et al., 2021; Siswanto et al., 2021). In the present study, in addition to the FAERS and JMDC databases, electronic medical records stored in the clinical data warehouse of Nihon University School of Medicine (NUSM’s CDW) containing detailed demographic, diagnostic, and laboratory data from patients at three hospitals affiliated with NUSM (Nagashima et al., 2022) were also used. Electronic medical records can provide continuous clinical information, leading to unattainably precise outcomes of the onset pattern of specific drug-induced adverse effects, treatment significance, and validated medication safety. To summarize, the corroborating results from the analysis of the three data sources mentioned above enable a more sensitive retrospective analysis for detecting causal relationships between the resultant adverse events and specific drug treatment, and the lowered incidence of adverse events and potential rescuing drugs causing this change. This hypothesis has been validated in animal experiments.

## Materials and methods

### Analysis of the FAERS database

Adversary event reports from 2004 to 2019 were downloaded from the FDA website (https://www.fda.gov/drugs/drug-approvals-anddatabases/fda-adverse-event-reporting-system-faers). Duplicate reports were deleted as previously reported (Banda et al., 2016), and 11,438,031 preserved reports were analyzed in the present study. Arbitrary drug names, including trade names and abbreviations, were manually mapped to unified generic names using the Medical Subject Headings (MeSH) descriptor ID. Reports of dyslipidemia were defined according to the preferred terms listed in Supplementary Table S1 of MedDRA (version 23.0). The FAERS data analysis was performed as previously described (Nagashima et al., 2016). Adversary event risk was evaluated by calculating the reporting odds ratio (ROR) along with the 95% confidence interval (CI) and *Z*-score (see Supplementary Tables S2, S3 for details). *Z*-scores were applied instead of *p*-values to save space graphically.

### Analyzing the JMDC claims database

Insurance claims data collected by JMDC Inc. from January 2005 to March 2018 were purchased, which contained the medical and prescription claims of 5,550,241 individuals and their dependents on a monthly basis. Health checkup data, including blood test results, body mass index (BMI), and waist circumferences, were provided by 2,278,697 individuals. The patients were mainly aged ≤ 65 years, and no patients aged ≥ 75 years were included. The drug names were coded using the Anatomical Therapeutic Chemical Classification System.

On retrospectively analyzing the profile of the first event onsets, the users of olanzapine were identified as those who were prescribed olanzapine more than 2 months after being included in the JMDC claims database. Patients without specific health checkup data were also excluded. Thus, 1,853 patients with olanzapine were included in the present study. Among them, vitamin D users were defined as patients whose first prescription of vitamin D was ahead of olanzapine treatment. The pre-prescription period for olanzapine was defined as the period within 12 months, while the post-prescription period was defined as the period within 12 months after initiating olanzapine treatment.

The blood test results included in the present study were triglyceride, LDL cholesterol, and high-density lipoprotein (HDL) cholesterol levels apart from BMI and waist circumstances. Test results were collected for each individual on the nearest date before initiating olanzapine treatment in the pre-prescription period and on the closest date in the post-prescription period after initiating olanzapine treatment. To reduce bias in population backgrounds, the propensity score matching method (greedy 1:1 matching) was used by balancing covariates between settings. The propensity score for vitamin D was obtained by fitting a logistic regression model that included all covariates of interest (Table 2). The propensity score of each patient with or without vitamin D treatment was subsequently matched using the nearest neighbor method. Using the matched outcomes, an unpaired two-tailed *t*-test with Welch’s correction for continuous variables and Fisher’s exact test for categorical data were conducted to compare the differences in baseline characteristics between patients with or without vitamin D exposure.

### Analysis of the NUSM electronic medical records

Electronic medical records were obtained from the Nihon University School of Medicine’s Clinical Data Warehouse (NUSM’s CDW). This database includes detailed diagnostic, demographic, and laboratory data for inpatients and outpatients. We received written informed consent and agreement for the secondary use after anonymization at three hospitals affiliated with the NUSM (Nagashima et al., 2022). Similar to the JMDC claims data analysis, the pre-prescription period of olanzapine was defined as the period within 12 months of the first prescription. Contrastingly, the post-prescription period was defined as the period within 12 months after initiating olanzapine treatment. Among them, preceding users of vitamin D were defined as patients whose first prescription of vitamin D was ahead of the first prescription of olanzapine for at least 1 day. Matching was not performed in this study because there were no significant differences in the baseline characteristics (Table 3). The blood laboratory values were extracted from the database, and the baseline value (month 0) was selected from the nearest test prior to the first prescription of olanzapine. The values at each checkpoint (months 3, 6, 9, and 12) were selected from the furthest test results from the first prescription of olanzapine. The missing values were imputed using the last observation carried forward method. An unpaired two-tailed *t*-test with Welch’s correction for continuous variables and Fisher’s exact test for categorical data were conducted to compare the differences in baseline characteristics between patients with or without vitamin D exposure.

### Animals

All animal experiments were approved by the Kyoto University Animal Research Committee in accordance with the ethical guidelines of the committee. All experiments were designed to minimize the use of animals and the number of required experiments. Male and female C57BL/6J mice were purchased from Japan SLC (Shizuoka, Japan) and housed at a constant ambient temperature (24 ± 1°C) and humidity (55% ± 10%) on a 12/12 h light/dark cycle. Mice were fed an *ad libitum* diet consisting of water and chow.

### Reagents and treatments (*in vivo*)

Olanzapine was purchased from the Tokyo Chemical Industry (Tokyo, Japan). The medium-fat diet (containing 1.37 IU of cholecalciferol/g) and cholecalciferol-supplemented medium-fat diet (containing 200 IU of cholecalciferol/g) were purchased from Oriental Yeast (Tokyo, Japan). Olanzapine was dissolved in water with 0.5% of carboxymethyl cellulose before use. Cholecalciferol is usually administered orally as a chow supplement, and the intake dose may vary slightly among mice. Mice were first randomized into two groups and fed a medium-fat diet or a cholecalciferol-supplemented medium-fat diet for 1 week. Mice from each group were further randomized to be treated with olanzapine (10 mg/kg, orally administered) or vehicle (0.5% of carboxymethyl cellulose solution) for another 5 days. One day after the last dose of olanzapine, the mice were anesthetized and dissected for blood collection by cardiac puncture. Mouse serum was isolated from blood samples by centrifugation. Serum total cholesterol levels were measured using a LabAssay Cholesterol kit (Wako, Osaka, Japan), while serum LDL cholesterol, HDL cholesterol, and triglyceride levels were measured by Nagahama Lifescience (Oriental Yeast Co., Ltd., Shiga, Japan).

### Cell culture

Primary mouse hepatocytes were prepared as previously described (Charni-Natan and Goldstein, 2020; Jung et al., 2020) with modifications. Livers from 6 to 8 weeks old male mice were perfused with Hanks’ balanced salt solution (HBSS) without calcium, magnesium, and phenol red, and subsequently supplemented with 0.5 mM of ethylenediaminetetraacetic acid (EDTA) and 25 mM of hydroxyethyl piperazineethanesulfonic acid (HEPES). The livers were then perfused with a digestive enzyme mix [collagenase, type I (Worthington, NJ, United States) 0.15 mg/mL; collagenase, type II (Worthington, NJ, United States) 0.15 mg/mL; Dispase, type II (Gibco, MA, United States) 0.15 mg/mL] solution, dissolved in standard HBSS with phenol red, and supplemented with 25 mM of HEPES. Hepatocytes were released into high-glucose Dulbecco’s modified Eagle’s medium (DMEM; D5796; Sigma-Aldrich, MO, United States) from digested livers, filtered through 70 μm cell strainers (Corning, NY, United States), and further purified using a 45% Percoll^®^ cushion by density gradient centrifugation. The purified cells were resuspended in William’s E medium (A1217601; Gibco) supplemented with 5% of fetal bovine serum (FBS; Sigma-Aldrich), 1 μM of dexamethasone (Nacalai Tesque, Kyoto, Japan), 1% of Penicillin–Streptomycin Mixed Solution (P/S; Nacalai Tesque), 5 μg/mL of human recombinant insulin (Sigma-Aldrich), 2 mM of GlutaMAX supplement (Sigma-Aldrich), and 15 mM of HEPES. Cells were plated onto dishes coated with 0.1 mg/mL of collagen type I (Nippi, Tokyo, Japan) and incubated at 37°C in a humidified chamber containing 5% CO_2_ for 3 h. After letting cells attach to the surface, the medium was refreshed with serum-free William’s E supplemented with 1 μM of dexamethasone, 0.5% of P/S, 1% of ITS + premix (Corning), 2 mM of GlutaMAX, and 15 mM of HEPES. Cells were used for treatment within 48 h.

Primary mouse adipocytes were prepared as previously described (Lequeux et al., 2009; Galmozzi et al., 2021) with modifications. Subcutaneous adipose tissues were collected from neonatal mouse pups and digested with 0.1 mg/mL collagenase type II and 0.1 mg/mL dispase type II dissolved in standard HBSS. Digested tissues were homogenized by pipetting, filtered through 70 μm cell strainers, and resuspended in high-glucose DMEM supplemented with 20% of FBS, 1% of P/S, and 10 mM of HEPES. The collected preadipocytes were plated onto dishes coated with 0.1% of gelatin (Nacalai Tesque) in distilled water. Cells were flushed twice with HBSS and refreshed with the same medium. After the cells reached 90% confluence, they were transferred into high-glucose DMEM supplemented with 10% of FBS, 1% of P/S, 10 mM of HEPES, 170 nM of human recombinant insulin, 1 μM of dexamethasone, 0.5 mM of 3-isobutyl-1-methylxanthine, 1 nM of triiodothyronine, and 10 nM of hydrocortisone for differentiation into adipocytes. After 48 h, the cells were transferred to high-glucose DMEM supplemented with 10% of FBS, 1% of P/S, 10 mM of HEPES, 170 nM of human recombinant insulin, and 1 nM of triiodothyronine. After 5 days of maintenance, the cells were ready for treatment.

Mouse C2C12 cells were prepared as previously described (Nagashima et al., 2016) with some modifications. The cells were obtained from Prof. H. Takeshima (Kyoto University Graduate School of Pharmaceutical Sciences, Kyoto, Japan). Cells were thawed in a water bath at 37°C and plated in high-glucose DMEM supplemented with 10% of FBS, 1% of P/S, and 10 mM of HEPES. After reaching 70% confluence, the cells were digested with 0.25% of trypsin solution (Nacalai Tesque) and passaged in the same medium. When the cells reached 90% confluence, the medium was changed to high-glucose DMEM supplemented with 2% of horse serum (Sigma-Aldrich), 1% of P/S, and 10 mM of HEPES for differentiation. The medium was refreshed 3 days after the changes, and the cells were ready on day five.

### Reagents and treatments (*in vitro*)

Calcifediol was purchased from Selleckchem (TX, United States) and calcitriol was purchased from Cayman (MI, United States. ZK159222 was purchased from Cayman. All reagents were reconstructed and preserved in DMSO as a vehicle at −80°C, and thus DMSO was added as a blank reference in control groups. Primary mouse hepatocytes were treated with maintenance medium. Primary mouse adipocytes and C2C12 cells were treated in Advanced DMEM/F12 medium (12634010, Gibco) supplemented with 1% of P/S, 2 mM of GlutaMAX supplement, and 10 mM of HEPES.

### RNA-seq and quantitative reverse transcriptase polymerase chain reaction (qRT-PCR)

RNA expression levels in cultured cells were evaluated using RNA-seq and qRT-PCR. Total RNA was isolated from cultured cells following the standard protocols provided by Isogen reagents (Nippon Gene, Tokyo, Japan).

For RNA-seq analysis, poly(A)^+^ RNA was selected from total RNA and sequenced using DNBseq (BGI, Shenzhen, China). Total reads were filtered by SOAPnuke (Cock et al., 2010) and clean reads were mapped to the mouse reference genome GRCm38.p6 using HISAT, version 2.0.4 (Kim et al., 2015) and Bowtie, version 2.2.5 (Langmead and Salzberg, 2012) in parallel. Gene expression was calculated using RSEM, version 1.2.8 (Li and Dewey, 2011). The gene set related to cholesterol biosynthesis was obtained from MGI (http://www.informatics.jax.org/vocab/gene_ontology/GO:0006695) (Hayamizu et al., 2005). All the genes from the three types of cultured cells (hepatocytes, adipocytes, and C2C12 cells) were mapped to this gene set to generate a list of cell-specific cholesterol biosynthetic process-related genes. Among the mapped genes, those with low expression levels (transcript per million (TPM) < 1) in vehicle-treated cells were removed from the analysis. Differentially expressed genes were detected between vehicle and olanzapine, olanzapine and olanzapine-calcifediol co-treated cells, and olanzapine and olanzapine-calcitriol co-treated cells based on PossionDis (Audic and Claverie, 1997). Genes with fold-change (FC) ≥ 2 and false discovery rate (FDR) ≤ 0.05 were recognized as differentially expressed. The analyzed results were visualized using volcano plots drawn using Prism 9.4.1 (GraphPad Software, CA, United States), with −log_10_ (FDR) in the y-direction and log_2_(FC) in the x-direction. The original sequence datasets were deposited in the NCBI sequence read archive with the accession number GSE221683.

For qRT-PCR, the extracted RNA was translated to cDNA using ReverTra Ace (Toyobo, Osaka, Japan) and subjected to StepOne real-time PCR (Life Technologies, Carlsbad, CA, United States) with Thunderbird SYBR qPCR Mix (Toyobo). The amplification process was set as follows: 10 min at 95°C, followed by 40 cycles of looping from 15 s at 95°C to 60 s at 60°C. Oligonucleotide primers were purchased from Sigma-Aldrich, and the sequences were as follows (gene name:5′-forward-3′, 5′-reverse-3′): Mus ribosomal protein, large, P0 (*Rplp0*): GCT TCG TGT TCA CCA AGG A, GTC CTA GAC CAG TGT TCT GAG G; Mus 3-hydroxy-3-methylglutaryl-CoA reductase (*Hmgcr*): GCT CGT CTA CAG AAA CTC CAC G, GCT TCA GCA GTG CTT TCT CCG T; Mus insulin-induced gene 2 (*Insig2*): GCC CAT CCA GAA CCT CTG AC, AGA CGG GGC AAA AGG ACT TC. The expression levels of each mRNA were normalized to those of *Rplp0* mRNA.

### Western blotting

Western blotting was performed as previously described (Ohashi et al., 2018) with some modifications. Cells were lysed in radioimmunoprecipitation assay (RIPA) buffer (Nacalai Tesque), diluted to 1 μg/mL with 1% sodium dodecyl sulfate (SDS) aqueous solution, and then buffered with NuPAGE^®^ lithium dodecyl sulfate (LDS) sample buffer (4×; Life Technologies, CA, United States). The samples were loaded onto a 10% SDS-polyacrylamide gel and blotted onto a ClearTrans^®^ PVDF Membrane (Wako). After blocking with Blocking One (Nacalai Tesque), the membranes were split into two pieces to simultaneously blot the target protein, vitamin D receptor (VDR), and the endo-reference protein, glyceraldehyde 3-phosphate dehydrogenase (GAPDH). The membranes were incubated overnight at 4°C with anti-VDR antibody (1:500 dilution, 12,550, Cell Signaling Technology, MA, United States) and anti-GAPDH (1:50,000 dilution, 32,233, Santa Cruz, CA, United States) in Tris-buffered saline supplemented with 0.1% of Tween 20 (TBS-T) and 10% of Blocking One. After washing with TBS-T, the membranes were incubated with peroxidase-conjugated donkey anti-rabbit IgG (1:5000 dilution, NA934V, GE Healthcare, IL, United States) and Peroxidase AffiniPure Goat Anti-Mouse IgG (1:5000 dilution, 115-035-003; Jackson ImmunoResearch, PA, United States) for 2 h at RT. Specific bands were detected using Immobilon Western Chemiluminescent HRP Substrate (Millipore) and visualized using EZCapture MG (ATTO, Tokyo, Japan). The expression level of VDR was normalized to that of GAPDH.

### Statistics

Statistical analysis of the FAERS database was performed using SQL Software (IBM Research Laboratory, NY, United States) and that of the JMDC database was performed using R version 3.5.1 Software (The R Foundation for Statistical Computing, Vienna, Austria). In the JMDC database, paired *t*-tests were used to compare the mean values within the pre-prescription and post-prescription periods. Differences in continuous variables between the two groups were compared using an unpaired two-tailed *t*-test with Welch’s correction. In the NUSM database, mixed-effects models were used to analyze repeated measures data.

Statistical analysis of the animal experiments was performed using GraphPad Prism 9.3.1, and the data are expressed as mean ± standard error of the mean (SEM). The blood profile data were analyzed using two-way analysis of variance (ANOVA) with *post hoc* Tukey’s multiple comparison test. Revalidation using qRT-PCR of the C2C12 cell line on RNA-seq resultant data was analyzed by two-way ANOVA. The dose-dependent changes in specific genes responding to calcifediol, calcitriol, and ZK159222 in the C2C12 cell line were analyzed by one-way ANOVA. The western blotting results of VDR cell-specific enrichment were analyzed using Student’s *t*-test. All reported *p*-values of < 0.05 were considered statistically significant.

## Results

### Analysis of FAERS

**FIGURE 1:**
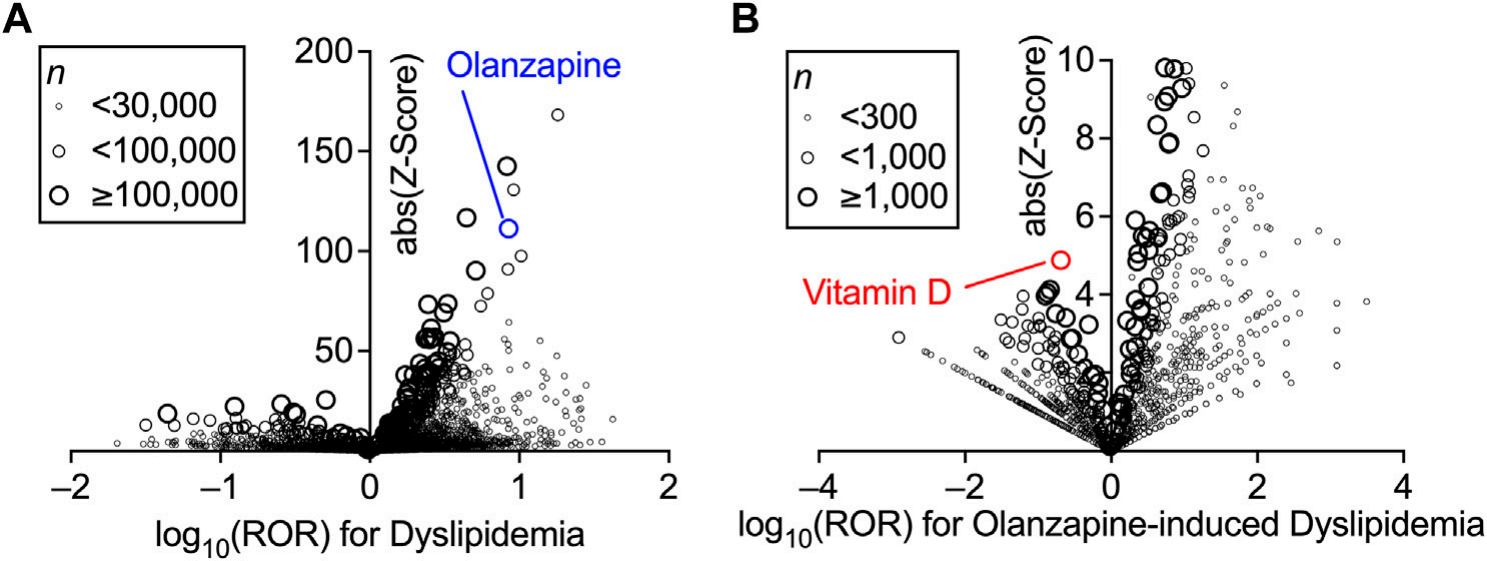
Increased incidence of dyslipidemia with the prescription of drugs and confounding effects of concomitant drugs on the olanzapine-induced dyslipidemia in FDA Adverse Event Reporting System (FAERS) data. Volcano plots for visualizing the reporting odds ratio (ROR, on a log scale) and its statistical significance (absolute *Z*-score) are shown. Each circle indicates an individual drug, and the size of the circle reflects the number of patients taking the drug. **(A)** Strong and significant increases in the ROR for dyslipidemia were seen in patients using olanzapine. **(B)** Within the population taking olanzapine, confounding effects of concomitantly used drugs on the incidence of olanzapine-induced dyslipidemia were calculated thoroughly and plotted. Overall values are presented in (Supplementary Tables S2, S3).

First, the association between the treatment with a particular drug and the incidence of dyslipidemia in the FAERS database was investigated using disproportionality analysis by calculating the ROR and *Z*-score of each drug (Figure 1A). The known reporting bias and lack of incidence denominators accompanied by self-reports (Alatawi and Hansen, 2017) only allowed these values to be limited demonstrations of the real-world incidence rate. Nevertheless, the high ROR and *Z*-score of olanzapine indicated that it was one of the drugs that exhibited the strongest association between its use and the onset of dyslipidemia (the complete set of data is provided in Supplementary Table S2). Olanzapine was selected for the present study because of its higher ROR than that of other atypical antipsychotics (Table 1).

**TABLE 1:** Association between atypical antipsychotics and the incidence of dyslipidemia in the FAERS data.

Drug A	Dyslipidemia with drug A	Dyslipidemia without drug A	ROR	*Z* score
Olanzapine	2,970/68,084 (4.36%)	60,845/11,369,947 (0.54%)	8.48	111.3
Quetiapine	3,309/123,599 (2.68%)	60,506/11,314,432 (0.53%)	5.12	90.3
Ziprasidone	770/18,224 (4.23%)	63,045/11,419,807 (0.55%)	7.95	56.0
Risperidone	1,804/98,718 (1.83%)	62,011/11,339,313 (0.55%)	3.39	50.6
Aripiprazole	1,509/80,484 (1.87%)	62,306/11,357,547 (0.55%)	3.46	47.2
Clozapine	933/67,960 (1.37%)	62,882/11,370,071 (0.55%)	2.50	27.6

The confounding effects of all drug combinations used in the olanzapine-treated patients were also calculated using the ROR and *Z*-score (Figure 1B). Vitamin D was found to be impressively effective in lowering the incidence of olanzapine-induced dyslipidemia. Although vitamin D was not among the drugs with the lowest ROR, which suggested higher effects on dyslipidemia, vitamin D had the highest *Z*-score due to its sufficient number of cases (the complete set of data is provided in Supplementary Table S3). However, vitamin D itself was associated with a slightly increased risk of dyslipidemia according to the FAERS data (ROR = 2.55, *Z*-score = 39.2; also see Supplementary Table S2), which may be due to the lack of time information to establish an accurate causal relationship between the reports and the effects of vitamin D. Thus, further analyses to reinforce the present findings were performed using the JMDC data.

### Analysis of JMDC insurance claims

**FIGURE 2:**
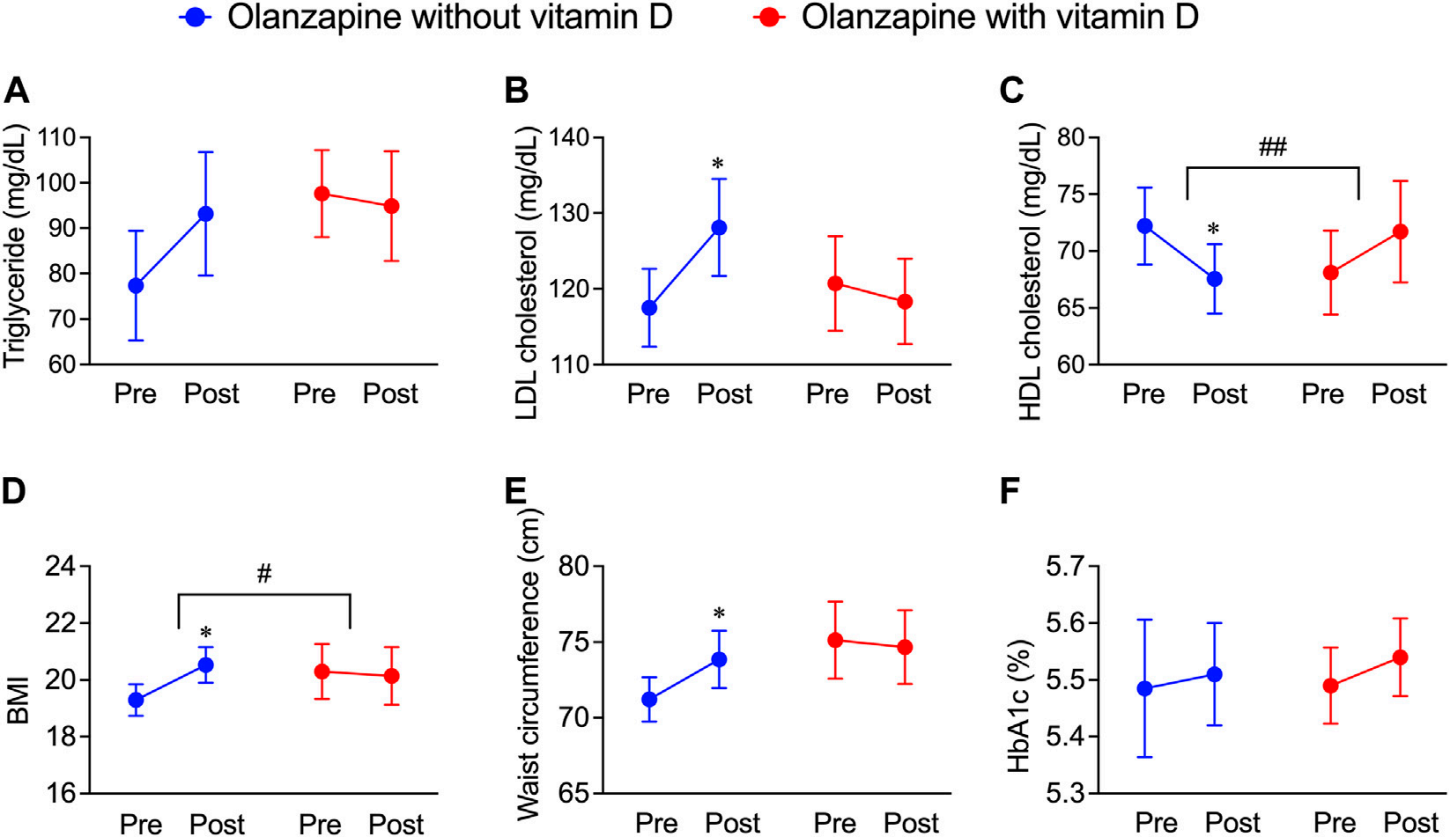
Deteriorative effects of olanzapine on blood lipid profiles and body weight maintenance and the rescuing effects of vitamin D co-treatment from the JMDC insurance claims. The data of pre-prescription and post-prescription periods were collected from the nearest test data before or after the olanzapine prescription within 1 year. Background characteristics were matched between groups (*n* = 20) as shown in Table 2. **(A–C)** The annual blood test results from patients included in JMDC were extracted. **(A)** Serum concentrations of triglycerides, **(B)** low-density lipoprotein (LDL) cholesterol, and **(C)** high-density lipoprotein (HDL) cholesterol were expressed in units of mg/dL. **(D,E)** The annual body weights, heights, and waist circumferences included in the JMDC were extracted. **(D)** Body mass index (BMI) is calculated from body weights and heights and **(E)** waist circumferences were obtained by direct measurement. **(F)** the level of HbA1c expressed in units of percentage of blood. Data are shown as means ± SEM. *p < 0.05; a paired *t*-test of the pre-prescription period against the post-prescription period. ^#^ *p* < 0.05, ^##^ *p* < 0.01; an unpaired two-tailed *t-*test with Welch’s correction between the group with and without vitamin D supplementation.

To further investigate whether the resultant dyslipidemia is associated with the clinical consequences of olanzapine treatment, the JMDC insurance claims data were introduced for analysis. Blood test data from the JMDC insurance claims of olanzapine-exposed 1,853 patients with or without vitamin D treatment were extracted (*n* = 22 for with vitamin D, *n* = 1,831 for without vitamin D). Since vitamin D is predominately prescribed to female elderly, a balancing approach, propensity score matching, was applied to eliminate possible systemic bias. After adjusting for populations, no significant differences in baseline characteristics between patients with or without vitamin D treatment (Table 2) were detected, and the batch size was balanced (*n* = 20). The effect of vitamin D on propensity score-matched data was investigated. In both groups of patients exposed only to olanzapine and to olanzapine and vitamin D simultaneously, the triglyceride level was not influenced (Figure 2A); however, the level of LDL cholesterol significantly increased by 11 mg/dL in the olanzapine-only group, while this value in patients with vitamin D co-treatment did not significantly increase (Figure 2B). Furthermore, the HDL cholesterol level in the blood significantly decreased by 5 mg/dL in the olanzapine-only group, while vitamin D supplementation significantly reversed the declining trend and even slightly increased HDL cholesterol levels by 4 mg/dL (Figure 2C). These results indicate that vitamin D counteracted the influence of olanzapine on blood lipid profiles.

**TABLE 2:** Population characteristics of the patients in the propensity score-matched olanzapine cohort extracted from the JMDC data; quantifiable data are given as means ± SEM.

	With vitamin D	Without vitamin D	*p*-value
Patients	20	20	NA
Elderly (≥65)	1 (5.0%)	1 (5.0%)	1
Female	18 (90.0%)	18 (90.0%)	1
Schizophrenia	4 (20.0%)	7 (35.0%)	0.48
Antipsychotics	6 (30.0%)	9 (45.0%)	0.51
Blood glucose (mg/dL)	91.9 ± 2.3	92.2 ± 3.1	0.94
HbA1c (%) (mg/dL)	5.49 ± 0.07	5.49 ± 0.12	0.97
Triglyceride (mg/dL)	97.6 ± 9.6	77.4 ± 12.0	0.2
LDL cholesterol (mg/dL)	120.7 ± 6.2	117.5 ± 5.1	0.69
HDL cholesterol (mg/dL)	68.1 ± 3.7	72.2 ± 3.4	0.42
BMI	20.3 ± 1.0	19.3 ± 0.6	0.38
Waist circumference (cm)	75.1 ± 2.5	71.2 ± 1.5	0.19

In addition to blood lipid profiles, the effects of vitamin D on body mass, fat distribution, and blood glucose levels in olanzapine-treated patients were investigated by analyzing related data from JMDC insurance claims. Within the same groups providing blood test data, BMI, an indicator used to roughly estimate whether one is overweight (as the higher BMI refers to a higher risk of being overweight), significantly increased by olanzapine treatment, while the co-treatment of vitamin D diminished this trend (Figure 2D). The mean value of the waist circumference, an approximate indicator of body fat mass, also significantly increased in olanzapine-only treated patients but not in olanzapine-vitamin D co-treated individuals (Figure 2E); however, the blood glucose indicator, HbA1c, was neither influenced in the olanzapine-only treated patients nor in the olanzapine-vitamin D co-treated individuals (Figure 2F). To summarize, these results indicate that using olanzapine aggravates the lipid profile, condition of being overweight, and excessive fat accumulation, while the combined use of vitamin D has beneficial effects on all three health aspects to some extent or at least provides no unfavorable effects. The blood and body test data in the JMDC insurance claims were annually collected from medical examinations provided by the Japan Health Insurance Association, which limited the precision of the data of the onset and development of olanzapine-induced dyslipidemia. Thus, to improve this flaw, electronic medical records were obtained from NUSM’s CDW, which is recorded in units of days, allowing tracing of blood profile changes on a smaller but more precise scale than that of the JMDC insurance claims data.

### Analysis of electronic medical records from NUSM’s CDW

**FIGURE 3:**
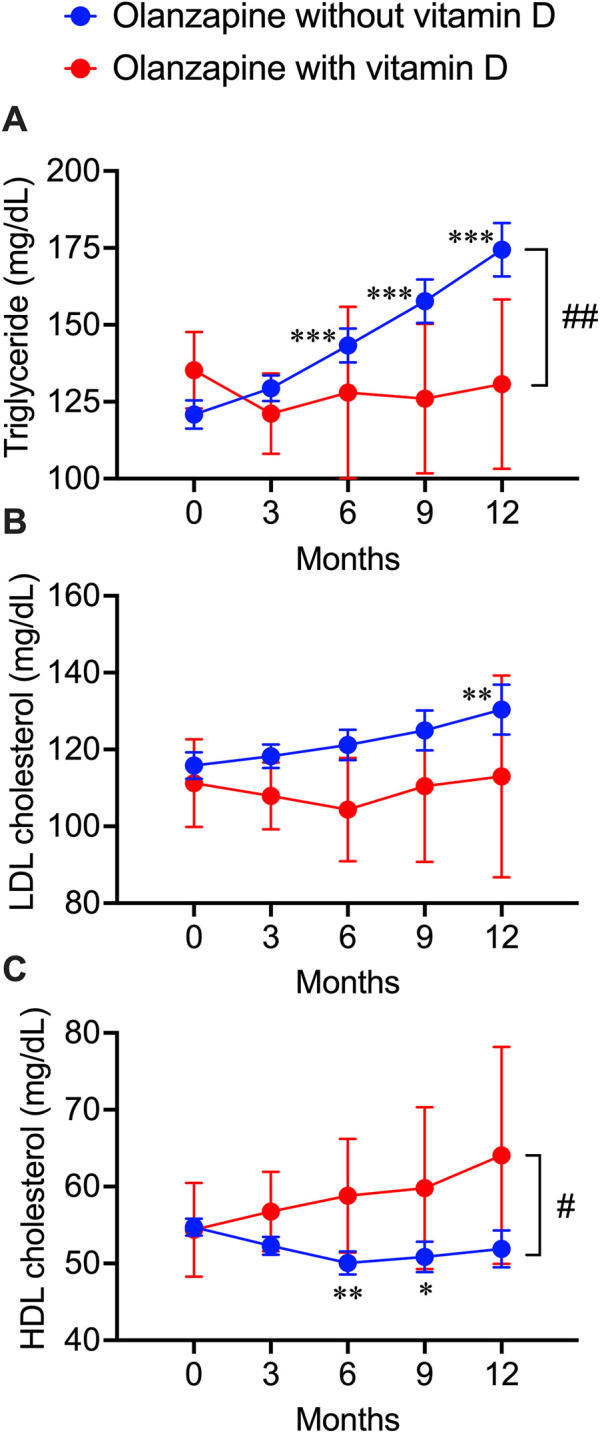
Deteriorative effects of olanzapine on blood lipid profiles and the rescuing effects of vitamin D co-treatment from electronic health records of the clinical data warehouse of Nihon University School of Medicine. Blood test results were extracted every 3 months after the first prescription of olanzapine. **(A)** the triglycerides (*n* = 406 in olanzapine without vitamin D group; *n* = 23 in olanzapine with vitamin D group), **(B)** the LDL cholesterol levels (*n* = 154 in olanzapine without vitamin D group; *n* = 11 in olanzapine with vitamin D group), and **(C)** the HDL cholesterol levels (*n* = 258 in olanzapine without vitamin D group; *n* = 16 in olanzapine with vitamin D group) were expressed in units of mg/dL. Data are shown as means ± SEM. *p < 0.05, **p < 0.01, ***p < 0.001; a paired *t*-test of data on any months after the olanzapine prescription against the data on month 0 (baseline). ^#^ *p* < 0.05, ^##^ *p* < 0.01; an unpaired two-tailed *t-*test with Welch’s correction between the groups with and without vitamin D supplementation.

The blood test data of patients exposed to olanzapine with or without vitamin D treatment, including TG, LDL cholesterol, and HDL cholesterol values, were obtained from the electronic medical records from NUSM’s CDW. No significant bias was detected between patients with and without vitamin D supplementation (Table 3). By continuously tracking the blood lipid profile changes within 1 year after the first exposure to olanzapine, a significant increase in the blood triglyceride level was detected as early as 6 months after initiating the treatment and kept increasing by 54 mg/dL at the end of the year, while co-treatment with vitamin D significantly diminished this trend and remained stable (Figure 3A). A significant increase in blood LDL cholesterol in patients without vitamin D supplementation was detected not earlier than 12 months from the first exposure to olanzapine and increased by 15 mg/dL, but this trend was not observed in patients with vitamin D supplementation (Figure 3B). Contrastingly, HDL cholesterol in the blood of patients without vitamin D supplementation significantly reduced by 5 mg/dL after 6 months of follow-up, while vitamin D supplementation especially prevented this risk from developing (Figure 3C). Based on NUSM’s CDW electronic medical recordings, the beneficial effects of vitamin D treatments on olanzapine-induced dyslipidemia can also be detected within a shorter time scale in contrast to the insurance claims from the JMDC.

**TABLE 3:** Population characteristics of the patients in the olanzapine cohort of the NUSM’s CDW database; quantifiable data are given as means ± SEM.

	With vitamin D	Without vitamin D	*p*-value
Patients	23	406	NA
Age (years)	57.1 ± 4.3	48.5 ± 0.9	0.06
Female	18 (78.3%)	249 (61.3%)	0.12
Schizophrenia	5 (21.7%)	110 (27.1%)	0.81
Antipsychotics	12 (52.2%)	225 (55.4%)	0.83
Blood glucose (mg/dL)	103.2 ± 4.7	107.6 ± 1.4	0.38
HbA1c (%)	5.55 ± 0.10	5.46 ± 0.04	0.39
Triglyceride (mg/dL)	135.2 ± 12.5	120.8 ± 4.6	0.29
LDL cholesterol (mg/dL)	125.5 ± 9.1	113.1 ± 2.9	0.21
HDL cholesterol (mg/dL)	52.9 ± 5.1	55.1 ± 1.0	0.67
BMI	Not available	Not available	NA
Waist circumference (cm)	Not available	Not available	NA

### Effects of vitamin D_3_ on olanzapine-induced dyslipidemia mouse model

**FIGURE 4:**
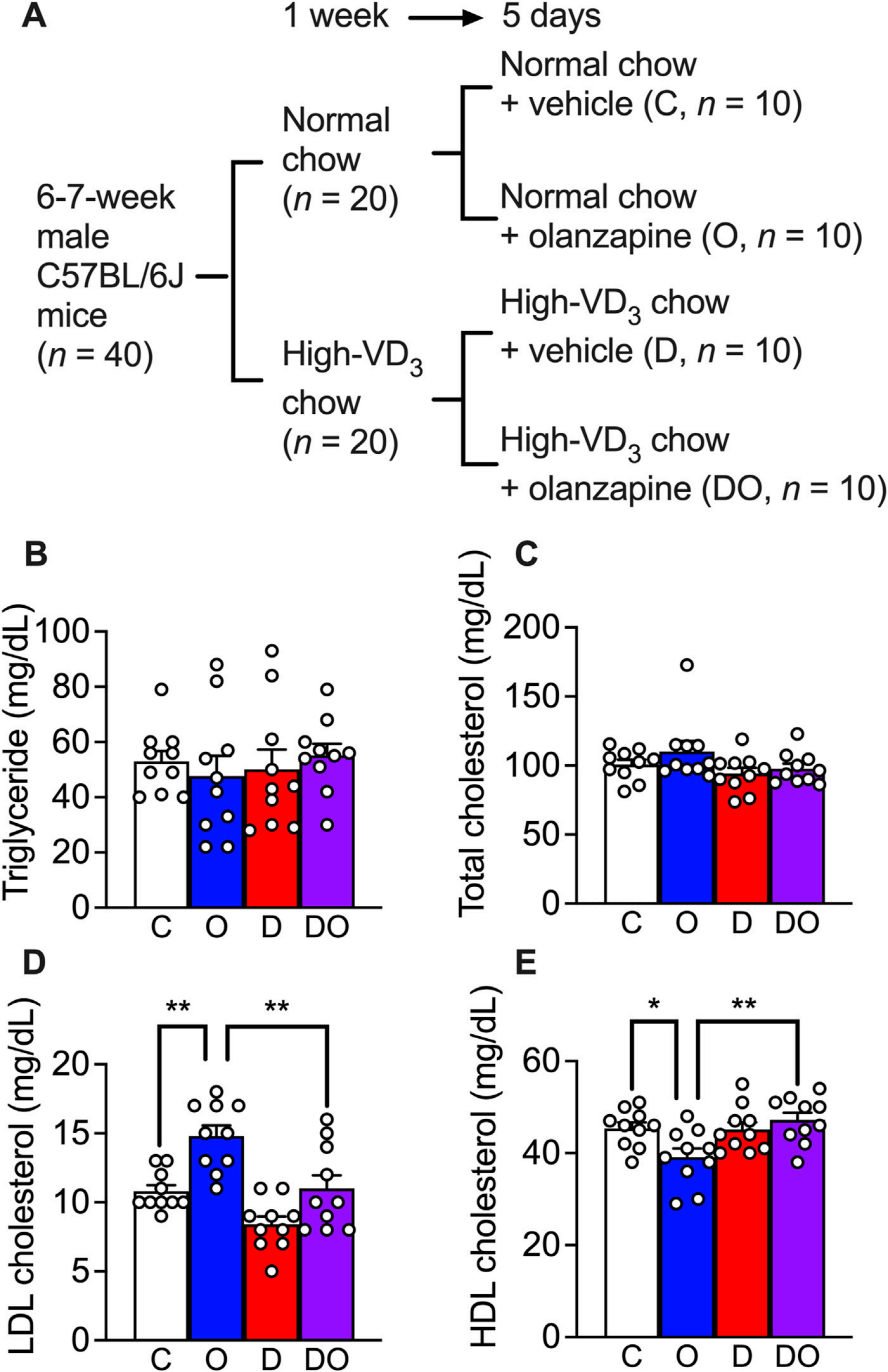
Olanzapine treatment caused dyslipidemia in a rodent model while vitamin D_3_ supplements attenuated the worsening of the condition. **(A)** C57BL/6J mice were fed for 1 week with the medium-fat diet (containing 1.37 IU of cholecalciferol/g of chow) or cholecalciferol-supplemented medium-fat diet (containing 200 IU of cholecalciferol/g of chow). Each group of mice were divided into two groups and orally given 10 mg/kg/day of olanzapine or vehicle for 5 days; resulting 4 groups of mice (each *n* = 10) received normal chow and vehicle as control **(C)**, normal chow and olanzapine (O), vitamin D_3_-supplemented chow and vehicle **(D)**, and vitamin D_3_-supplemented chow and olanzapine (DO). Blood samples were collected from these mice 1 day after the last dose of olanzapine without fasting. **(B)** the triglycerides, **(C)** total cholesterol levels, **(D)** LDL cholesterol levels, and **(E)** HDL cholesterol levels were measured. Data are shown as means ± SEM. The multiple comparisons of olanzapine and cholecalciferol influences were compared by two-way analysis of variance (ANOVA) with *post hoc* Tukey’s multiple comparison test. *p < 0.05, **p < 0.01.

Previous studies have reported non-alcoholic fatty liver disease and LDL cholesterol or HDL cholesterol imbalance in the blood phenotypes of olanzapine-treated wild-type mouse models (Chen et al., 2018; Jiang et al., 2019; Liu et al., 2019; Zhu et al., 2022) under chronic treatment (at least 4 weeks). In the present study, in order to study the primary stage of dyslipidemia development, the treatment was limited to 5 days (Figure 4A). To mimic the calorie intake of human patients, mice were provided with 30% of fructose in drinking water during the acclimatization period of 1 week and this was continued in the following runs of treatment (Faeh et al., 2005). The results showed that neither a scheduled administration of olanzapine (10 mg/kg, orally administered by gavage, daily for 5 days) nor vitamin D supplementation (administrated *via* a cholecalciferol (vitamin D_3_)-supplemented diet containing 200 IU of cholecalciferol/g) caused changes in blood triglyceride (Figure 4B) and total cholesterol levels (Figure 4C). However, a significant increase in the blood LDL cholesterol level and the attenuating effects of vitamin D supplementation were detected (Figure 4D). In addition, the reduction in HDL cholesterol level caused by olanzapine treatment was attenuated by cholecalciferol supplementation (Figure 4E). These findings suggest that short-term administration of olanzapine is sufficient to induce dyslipidemia by challenging the balance between blood LDL and HDL cholesterol levels without influencing blood triglyceride and total cholesterol levels. Simultaneously, cholecalciferol as dietary supplementation diminished these effects.

### Molecular mechanisms of vitamin D on olanzapine-induced dyslipidemia

**FIGURE 5:**
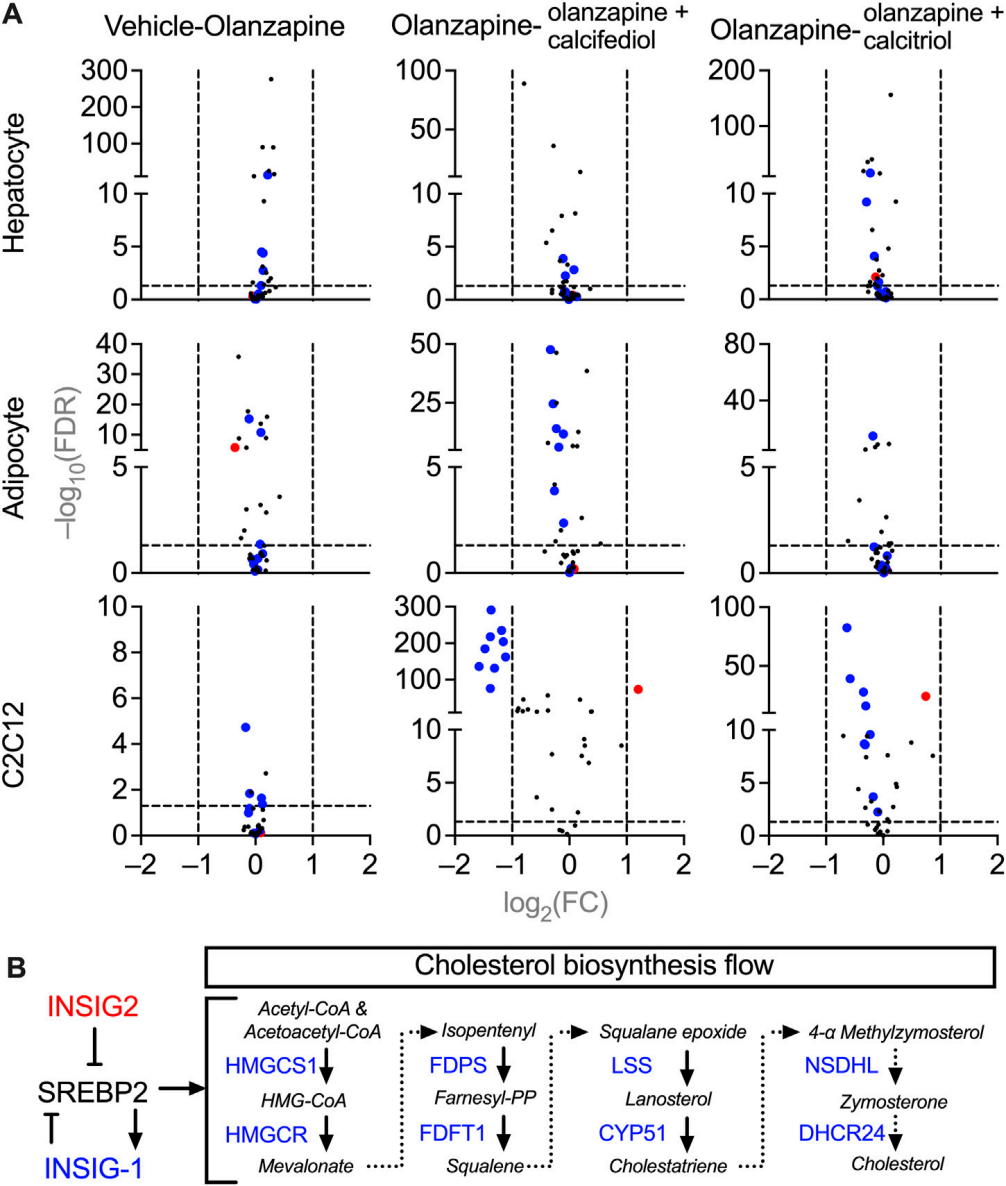
Volcano plots of gene expression in the cultured mice cells treated with vehicle, olanzapine, or olanzapine co-treated with calcifediol or calcitriol. The changing ranges were expressed as log_2_-transformed fold-change (FC), and the significance was expressed as −log_10_-transformed false discovery rate (FDR). **(A)** Demonstrations of the cholesterol biosynthesis-related gene changes under olanzapine treatment and calcifediol or calcitriol-olanzapine co-treatment. The genes significantly downregulated in the olanzapine-calcifediol co-treated C2C12 cells are tagged with blue dots, and the genes upregulated are tagged with red dots in all the data sets. **(B)** A schematic diagram illustrating the functions of downregulated genes (blue) and upregulated gene (red) in the process of cholesterol biosynthesis. Genes plotted with FC absolute value > 2 and FDR value > 0.05 were considered to be significantly changed by treatment.

To investigate the molecular mechanisms underlying cholesterol dyshomeostasis in blood accompanied by sub-chronic olanzapine and the rescuing effects of cholecalciferol supplementation, changes in expression of genes in cells related to cholesterol metabolism were analyzed using RNA-seq. Purified primary cultures of mouse hepatocytes, adipocytes, and fully differentiated C2C12 cells (myotubes) were used instead of liver, adipose, and skeletal muscle tissues, since these tissues are generally the mixtures of various cells with different origins and functions, which may blur the direct effects of olanzapine and vitamin D metabolites. All three types of cells were treated under the same condition, a 24 h pre-exposure to the circulating form of vitamin D_3_, 25-hydroxycholecarciferol, (calcifediol at 10 μM) or the active form of vitamin D_3_, 1,25-dihydroxycholecarciferol, (calcitriol at 0.1 μM), followed by another 12 h co-treatment with olanzapine (1 μM). From the MGI database, only cholesterol biosynthesis-related genes were selected for analysis (Hayamizu et al., 2005) because only cholesterol homeostasis was disrupted in the blood according to *in vivo* modeling. Comparing these gene expressions under different treatments in various types of cells with PossionDis (Audic and Claverie, 1997), the expression of a set of genes was significantly changed in olanzapine-calcifediol-treated C2C12 cells and slightly influenced in olanzapine-calcitriol-treated C2C12 cells. However, olanzapine displayed passive effects in all three cell types (Figure 5A). The regulatory relationship within this set of genes is illustrated in Figure 5B. In this scheme, *Insig2*, a suppressive regulator of cholesterol biosynthesis (Yabe et al., 2002), was upregulated by vitamin D metabolites, while genes participating in the mevalonate pathway (DeBose-Boyd, 2008), such as 3-hydroxy-3-methylglutaryl-CoA reductase (*Hmgcr*), 3-hydroxy-3-methylglutaryl-coenzyme A synthase 1 (*Hmgcs1*), farnesyl diphosphate synthetase (*Fdps*), farnesyl diphosphate farnesyl transferase 1 (*Fdft1*), lanosterol synthase (*Lss*), cytochrome P450, family 51 (*Cyp51*), NAD(P) dependent steroid dehydrogenase-like (*Nsdhl*), and 24-dehydrocholesterol reductase (*Dhcr24*) were significantly downregulated.

**FIGURE 6:**
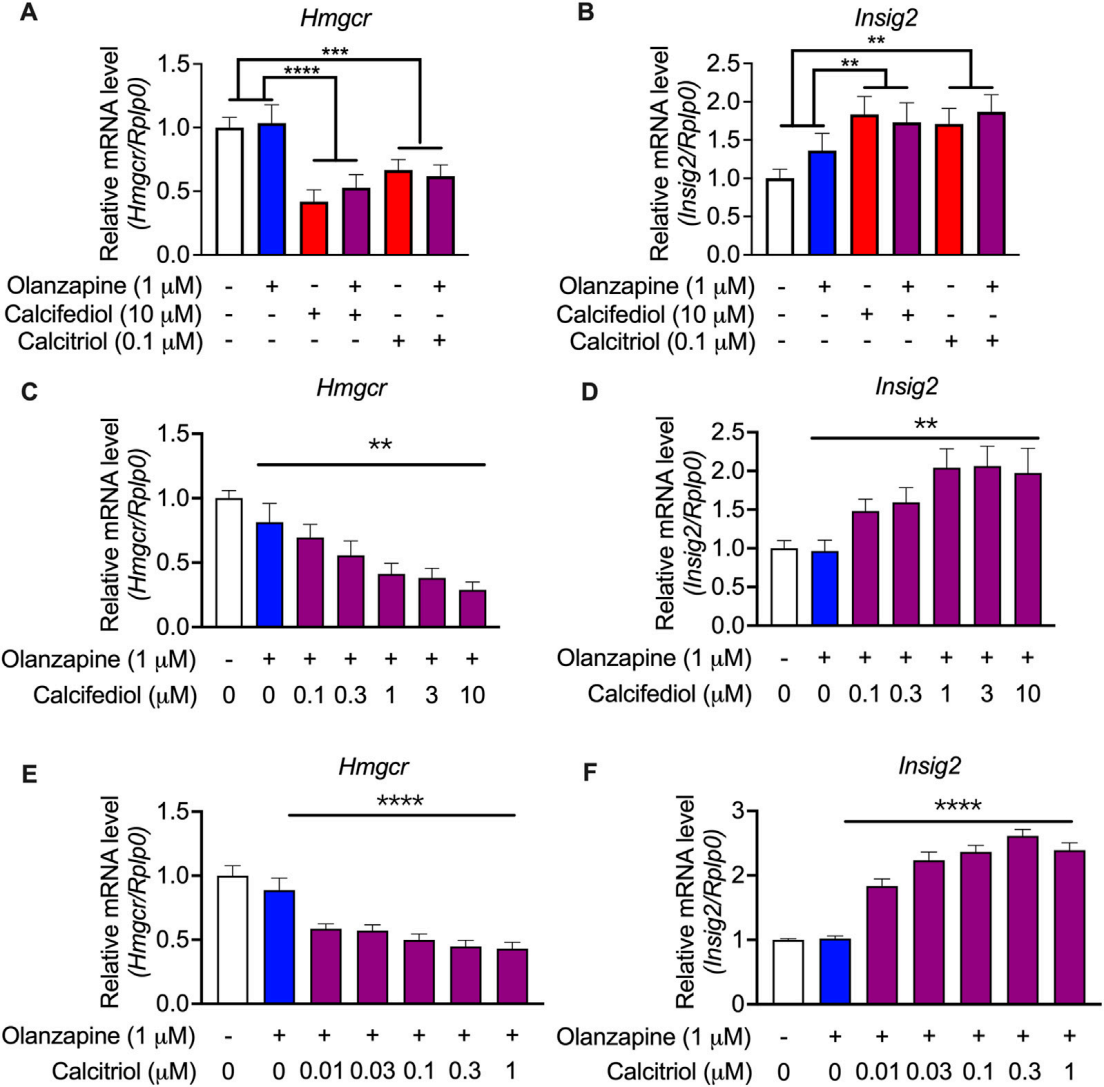
Validation by quantitative RT-PCR results showing the expression of cholesterol biosynthesis-associated genes in the cultured C2C12 cell line. **(A)** 3-hydroxy-3-methylglutaryl-CoA reductase (*Hmgcr*) and **(B)** Insulin-induced gene 2 (*Insig2*) were validated in the same treating condition as that in the RNA-seq experiments (*n* = 12). Results were normalized to ribosomal protein, large, P0 (*Rplp0*). **(C)** *Hmgcr* and **(D)** *Insig2* were validated in calcifediol with gradient-changed concentrations increasing from 0 to 10 μM (*n* = 8–9). **(E)** *Hmgcr* and **(F)** *Insig2* were validated in calcitriol with gradient-changed concentrations increasing from 0.01 μM to 1 μM. Data are shown as means ± SEM (*n* = 9). **p < 0.01, ****p < 0.0001. The comparisons of olanzapine and vitamin D influences were compared by two-way analysis of variance (ANOVA). The validation of the dose-dependent effects of calcifediol and calcitriol were compared by one-way ANOVA.

To validate these findings from the RNA-seq analysis, an enlarged batch of evaluations was performed on C2C12 cells, which displayed the highest sensitivity to vitamin D metabolites, using qRT-PCR. *Hmgcr* was selected as a representative gene for cholesterol biosynthesis because it is the most representative gene involved in the process of cholesterol biosynthesis (Horton et al., 2003). The qRT-PCR results revealed that both the suppression of *Hmgcr* expression (Figure 6A) and the induction of the expression of *Insig2* (Figure 6B) by calcifediol and calcitriol were significant. A previous report claimed that calcifediol, but not calcitriol, independently reduced cholesterol biosynthesis without VDR actions (Asano et al., 2017). However, further validations by changing the concentration of either calcifediol or calcitriol in the treatment medium showed that the expression of *Hmgcr* (Figure 6C) and *Insig2* (Figure 6D) changed in a dose-dependent manner under calcifediol treatment. In parallel, the changes in the expression of *Hmgcr* (Figure 6E) and *Insig2* (Figure 6F) also displayed a dose-dependent effect in calcitriol treatment, with approximately 5.8 times higher sensitivity (IC_50_ of calcifediol on Hmgcr: 0.68 µM; IC_50_ of calcitriol on Hmgcr: 0.12 µM). Since calcitriol is the bioactive form of vitamin D, which acts only through VDR, the role of VDR in cholesterol synthesis suppression in this model should not be underestimated and requires further investigation.

**FIGURE 7:**
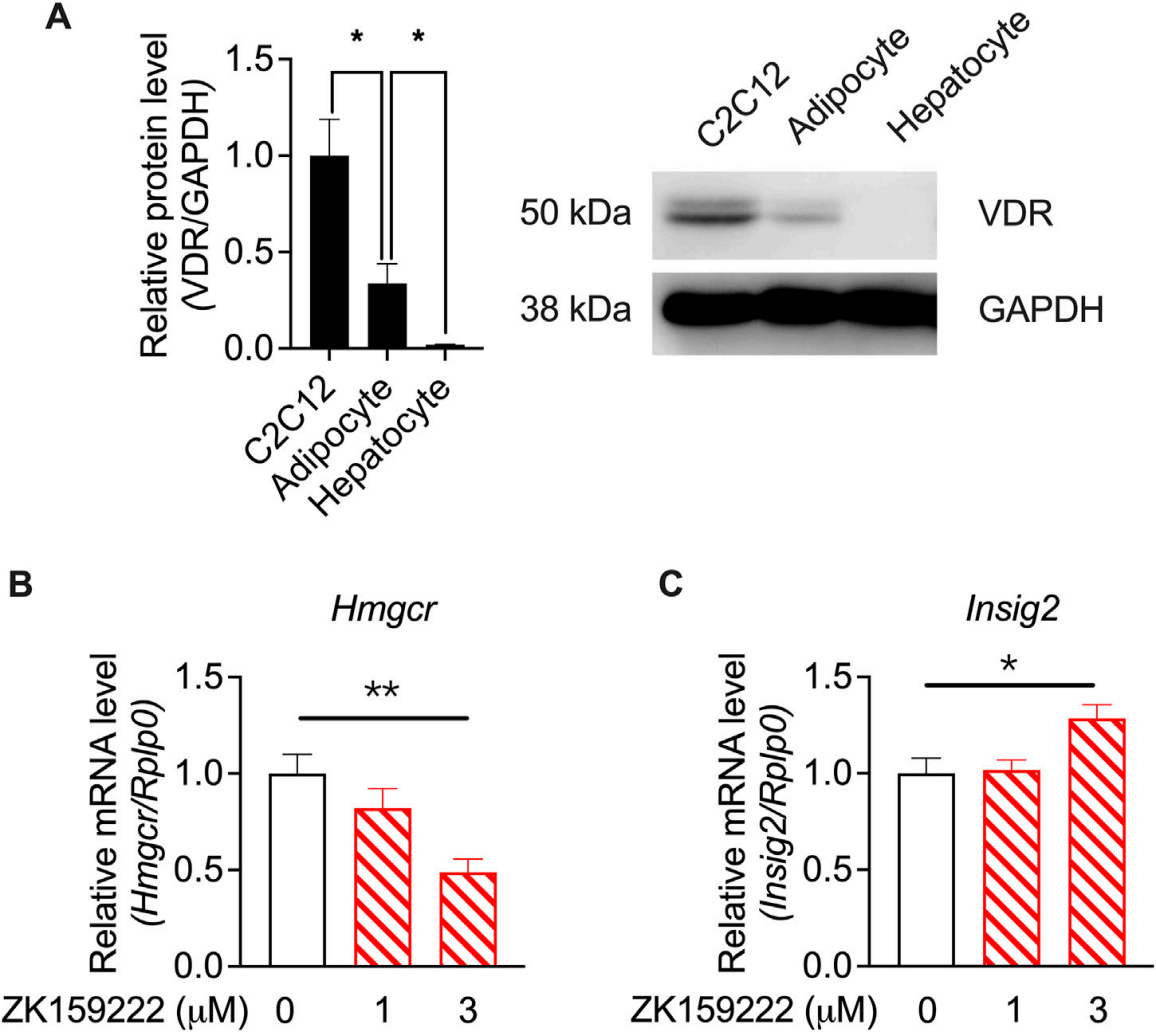
Validation by quantitative RT-PCR results showing calcitriol regulating the cholesterol biosynthesis-associated genes mediated by VDR in the C2C12 cell line. **(A)** The results of vitamin D receptor (VDR) protein levels in C2C12 cells, adipocytes, and hepatocytes, normalized to glyceraldehyde 3-phosphate dehydrogenase (GAPDH) (*n* = 3). **(B)** *Hmgcr* and **(C)** *Insig2* were validated in ZK159222 with gradient-changed concentrations increasing from 0 to 3 μM (*n* = 6). Data are shown as means ± SEM. *p < 0.05, **p < 0.01. The validation of the dose-dependent effects of ZK150222 was compared by one-way analysis of variance (ANOVA).

To investigate whether the actions of calcifediol and calcitriol were mediated by VDR activity, western blotting of VDR levels in specific cell types was performed using RNA-seq. VDR was highly expressed in C2C12 cells, which was much higher than that in adipocytes and hepatocytes (Figure 7A). The varied tissue-specific enrichment of VDR indicates that suppression of *Hmgcr* expression is mediated by VDR activity. A partial agonist of VDR (Teske et al., 2016), ZK159222, was used to validate the action of VDR. ZK159222, like calcifediol and calcitriol, suppressed *Hmgcr* expression (Figure 7B) and induced *Insig2* expression (Figure 7C) in a dose-dependent manner, further confirming the essential role of VDR activity.

## Discussion

In this study, we showed for the first time that vitamin D supplementation is effective in improving the lipid profiles in patients treated with olanzapine. This finding was revealed from three independent sources of clinical data. This hypothesis was validated by using the blood profiles of mice models fed with high-calorie diets, and the mechanism of action was investigated at the molecular level using cells that were metabolically active with cholesterol.

Drug repurposing using FAERS has been proven to be a reliable tool for refining unexpected drug-drug interactions with beneficial effects in treating adverse effects (Nagaoka et al., 2021; Siswanto et al., 2021). In the present study, olanzapine was one of the most dyslipidemia-inducing drugs causing a large ROR in more than tens of thousands of cases, according to the FAERS database, providing a solid foundation to further explore rescue drugs. Additionally, olanzapine had a greater tendency to induce severe dyslipidemia than other atypical antipsychotics that share similar molecular structures, such as clozapine and quetiapine, in the FAERS database (Table 1). Notably, patients are more heavily dosed with clozapine (C_max_ > 1.07 μM) (Jann et al., 1993) and quetiapine (C_max_ ≈ 0.21 μM) (DeVane and Nemeroff, 2001) than olanzapine (C_max_ < 0.064 μM) (Callaghan et al., 1999) in clinical practice. These two facts prioritize screening drug targets for treating olanzapine-induced dyslipidemia from other atypical antipsychotics. Contrastingly, vitamin D treatment is recognized as a dyslipidemia risk factor other than olanzapine, which is likely caused by the confounding in identifying causes and results. Since patients with dyslipidemia are treated with this drug, the onset of the disease as per the FAERS data, is also likely to be positively related to the usage of this drug. This confusion is due to the lack of information regarding the prescription period to help define the time point of drug exposure and disease onset. However, cross-validations using health databases with records on prescription time can help solve this issue.

The hypothesis developed from the FAERS data analysis was verified in a retrospective cohort analysis based on the JMDC insurance claims data. The onset rate of dyslipidemia and the drug exposure period can be analyzed in units of years using the raw blood test results of patients under 75 years of age that the JMDC claims can annually provide as a supplement to the FAERS data. In the present study, propensity score matching revealed olanzapine-induced dyslipidemia and weight gain in the matched groups. However, the patient population characteristics are strongly sex-biased (primarily women), because vitamin D is predominantly prescribed to women in cases where they are more susceptible to osteoporosis (Bohon and Goolsby, 2013). Nevertheless, olanzapine has been reported to increase LDL cholesterol and triglyceride levels in adolescents (Kryzhanovskaya et al., 2009) and young adults (Narula et al., 2010), regardless of gender variance, indicating that the analysis results from JMDC claims still remain highly consistent with clinical trials. The present study found that vitamin D supplementation during the exposure period to olanzapine suppressed the increase in the LDL cholesterol level, decrease in the HDL cholesterol level, increase in BMI, and increase in waist circumference. This is the first study to demonstrate the clinical significance of vitamin D in improving blood lipid profiles, since only one previous trial has shown that vitamin D helps in maintaining waist circumference in olanzapine-treated patients (Kaviyani et al., 2017). Despite already being efficient in discovering the olanzapine-vitamin D interaction at the laboratory test level, the JMDC is still unable to precisely locate the onset time pattern of olanzapine-induced dyslipidemia due to the long data collection intervals. Further improvement was achieved by utilizing the electronic medical records from NUSM’s CDW, since the electronic records were updated daily and allowed for a flexible time-course setting. The blood test results were collected every 3 months after initiating the olanzapine treatment. The triglyceride and LDL cholesterol levels were reported to increase monotonically as the exposure time increased, and the appearance of abnormality in triglyceride levels was prior to that in the LDL and HDL cholesterol levels. Differentiating from JMDC claims, patients treated with olanzapine only exhibited a strong trend in developing triglyceride abnormality, while triglyceride levels from patients in JMDC claims were passively influenced. This inconsistency may be due to the variance in population background characteristics, since patients included in the electronic records from NUSM’s CDW patients were more balanced in terms of sex (female percentage = 61.3%) than those from the JMDC claims (female rate = 90.0%), and higher estrogen levels in females are generally believed to be the reason for better triglyceride control than males (Carr, 2003). Vitamin D treatment counteracted these deteriorative changes by maintaining the blood lipid profile at stable levels throughout the observation window, consistent with the outcomes of the JMDC claims. By combining these three outcomes based on different data sources, a causal relationship between lipid profiles’ deterioration and olanzapine treatment was detected in the present study, and the suppressive effects of vitamin D supplementation on these influences were also confirmed.

Using drug-induced animal models, significant efforts have been devoted to identifying the mechanism of olanzapine-induced dyslipidemia. In several rodent models, strong hepatoxicity, represented by non-alcoholic fatty liver diseases, has generally been reported after chronic treatments ranging from 4 to 12 weeks (Chen et al., 2018; Jiang et al., 2019; Liu et al., 2019; Zhu et al., 2022). However, these models cannot accurately represent clinical observations from patients, since olanzapine rarely causes clinically apparent hepatotoxicity (Larrey and Ripault, 2013). Furthermore, it generally takes years for non-alcoholic fatty liver disease to develop under constant environmental stress (Friedman et al., 2018). Contrastingly, according to the electronic medical records from NUSM’s CDW in the present study, the onset of dyslipidemia was as early as 6 months after initiating olanzapine treatment, which suggests that the development of olanzapine-induced dyslipidemia is not necessarily related to the onset of hepatic diseases. This assumption is supported by the *in vivo* observation in mouse models in the present study that olanzapine exposure as short as 1 week is already enough to cause the LDL and HDL cholesterol levels to increase and drop, respectively. A recent clinical study revealed an alternative explanation to olanzapine-induced dyslipidemia that an increased appetite is essential for developing olanzapine-induced dyslipidemia (Huang et al., 2020), and moreover, enhanced food reward circuitry has been detected in olanzapine-treated patients (Mathews et al., 2012). This phenotype was previously observed in rodent models and attributed to the antagonistic effects on histamine H_1_ receptors in the central nervous system by olanzapine (Kim et al., 2007; He et al., 2014). Nevertheless, a slight increase in the expression of genes related to cholesterol biosynthesis was detected in the hepatocytes in the present study. However, the changing ranges were too limited to be considered investigation-worthy. In conclusion, hepatotoxicity is not causally related to olanzapine-induced dyslipidemia and the direct influence of olanzapine on lipid metabolic processes in related tissues lacks physiological significance.

However, the role of vitamin D in lipid metabolism remains controversial. Several studies have shown that vitamin D is necessary for lipid synthesis and accumulation in adipose tissue. In VDR-knockout mouse models, the absence of VDR showed substantial protective effects against high-fat diet-induced adipose tissue accumulation (Narvaez et al., 2009; Wong et al., 2009), whereas in another knock-in model, mice with overexpressed human VDR in adipose tissue developed obesity even under a standard diet (Wong et al., 2011). Further investigation into this mechanism revealed that vitamin D potentiates subcutaneous preadipocyte differentiation *via* the VDR pathway. Simultaneously, the expression level of VDR significantly decreased as differentiation progressed until the full maturity of adipocytes (Nimitphong et al., 2012). Contrastingly, vitamin D alleviated atherosclerosis progression in patients by preventing foam cell formation (Oh et al., 2009) and suppressing oxidative stress in blood vessel endothelial cells (Kassi et al., 2013). However, the role of vitamin D in lipid homeostasis is not necessarily associated with maintaining the blood lipid profile. Clinical observations have reported improved blood triglyceride and LDL cholesterol levels in populations supplied with extra vitamin D, with varied background characteristics, according to two meta-analyses (Mirhosseini et al., 2018; Dibaba, 2019). The big data mining-based results in the present study are consistent with these findings. However, according to a previous study on the effects of calcium supplements on the blood lipid profile, the primary medical function of vitamin D, which improves calcium absorption, is not likely to be related to its role in treating dyslipidemia (Reid et al., 2010). To date, studies revealing the mechanisms of these clinical results are lacking. Nevertheless, a recent study established an inhibitory connection between vitamin D and lipid synthesis by proving that calcifediol, but not calcitriol, inhibits cholesterol and fatty acid biosynthesis by preventing sterol regulatory element binding transcription factors from maturing in a VDR-independent pattern (Asano et al., 2017). However, this result was not reproduced in primary hepatocytes and adipocytes, which may be due to the vast metabolic variance between highly differentiated primary cells and immortalized cells in that report (CHO cell line). Another study suggested that VDR-mediated *Insig2* expression by calcitriol in HepG2 cells mainly contributed to its suppressive effects on cholesterol synthesis (Li et al., 2016). This result is doubtful because VDR is absent in normal hepatocytes (Gascon-Barre et al., 2003). This understanding is consistent with the lack of VDR expression in primary cultured hepatocytes in the western blot results of the present study. Nevertheless, the VDR activation-induced *Insig2* expression increase is reproduced in this study. Insig2 is an essential regulator of cholesterol biosynthesis that not only prevents sterol regulatory element binding transcription factors from entering the cell nucleus to suppress their transcription function (Yabe et al., 2002) but also facilitates HMGCR degradation (Jo et al., 2011). A functional vitamin D response element has been localized in the promoter of the mouse *Insig2* (Lee et al., 2005), and further comparisons between rodent and human *Insig2* promoters have confirmed that this vitamin D response element is also included in the human genome (Fernandez-Alvarez et al., 2010). Although in contrast to the liver, the role of the muscle tissues is minor in the essentiality of cholesterol production and balance, cholesterol production in peripheral tissues should not be overlooked. A report has shown myopathy development in skeletal muscle-specific *Hmgcr* knockout mice (Osaki et al., 2015). Additionally, humans with skeletal muscle *Hmgcr* deficiency easily develop myopathy (Lorenzo-Villalba et al., 2021). Furthermore, as VDR is widely expressed in tissues throughout the body, vitamin D may improve the blood cholesterol condition by limiting tissue self-biosynthesis of cholesterol and simultaneously inducing cholesterol absorption in peripheral tissues. A primary limitation of this research is the failure to clarify the mechanism of olanzapine-induced dyslipidemia through investigations on lipid metabolism. Future explorations will focus on the neurological role of olanzapine in increasing appetites. Additionally, the investigation on vitamin D target tissues and organs is also limited, since the enterocytes in the intestine are also essential for lipid absorption and highly enriched with VDR. The possible role of enterocytes is also scheduled for future studies.

Although the exploration of the potential of VDR-mediated cholesterol biosynthesis suppression in new druggable target development still has a long way to cover, from the clinical findings in this research, it is safe to suggest vitamin D supplements in patients under olanzapine treatment, especially in those diagnosed as vitamin D deficient. Moreover, this treatment is also recommended to be simultaneously applied with a calorie-limited diet for patients under olanzapine treatment for a better outcome.

## Data Availability

The datasets presented in this study can be found in online repositories. The names of the repository/repositories and accession number(s) can be found below: NCBI Gene Expression Omnibus (GEO) [https://www.ncbi.nlm.nih.gov/geo/], GSE221683.
