# Supplementary material for: Vitamin D supplementation is effective for olanzapine-induced dyslipidemia
Source: Front Pharmacol. 2023 Feb 21;14:1135516. doi: 10.3389/fphar.2023.1135516 (PMC9989177; doi:10.3389/fphar.2023.1135516)
Supplement: Supplementary file 1 [file DataSheet1.docx]

**Supplementary materials**

**Supplementary Table S1** Search terms for dyslipidemia in FAERS database

|  | | | |
| --- | --- | --- | --- |
| Blood cholesterol increased | Blood triglycerides increased | Hyperlipidaemia | Hypercholesterolaemia |
| Low density lipoprotein increased | Hypertriglyceridaemia | Dyslipidaemia | High density lipoprotein decreased |
| Blood cholesterol abnormal | Blood cholesterol decreased | Lipids increased | Low density lipoprotein decreased |
| High density lipoprotein increased | Blood triglycerides abnormal | Lipids abnormal | Low density lipoprotein abnormal |
| Type V hyperlipidaemia | Blood triglycerides decreased | Lipid metabolism disorder | High density lipoprotein abnormal |
| Very low density lipoprotein increased | Total cholesterol/HDL ratio increased | Non-high-density lipoprotein cholesterol increased | Lipoprotein (a) increased |
| Total cholesterol/HDL ratio decreased | Type IIa hyperlipidaemia | Lipids decreased | Total cholesterol/HDL ratio abnormal |
| LDL/HDL ratio increased | Hypo HDL cholesterolaemia | Very low density lipoprotein decreased | Type IIb hyperlipidaemia |
| Type IV hyperlipidaemia | Very low density lipoprotein abnormal | LDL/HDL ratio decreased | Lipoprotein (a) abnormal |
| Diabetic dyslipidaemia | Acquired mixed hyperlipidaemia | Blood cholesterol esterase increased | Type III hyperlipidaemia |
| Familial hypertriglyceridaemia | Hypotriglyceridaemia | Intermediate density lipoprotein increased | Non-high-density lipoprotein cholesterol decreased |
| Type II hyperlipidaemia | Remnant-like lipoprotein particles increased | Fat overload syndrome | Apolipoprotein B/Apolipoprotein A- 1 ratio increased |
| Primary hypercholesterolaemia | Lecithin-cholesterol acyltransferase deficiency | Remnant hyperlipidaemia | Acquired lipoatrophic diabetes |
| Intermediate density lipoprotein decreased | Type I hyperlipidaemia | Autoimmune hyperlipidaemia | Cardiometabolic syndrome |
| Lipoprotein (a) decreased | Lipoprotein abnormal | Familial high density lipoprotein deficiency | Lipoprotein increased |
|  | | | |

Supplementary Data.xlsx: Table S2

**Supplementary Table S2** Overall results of disproportionality analysis for dyslipidemia in the FDA Adverse Event Reporting System (FAERS) data

Individuals in the FAERS data were divided into the following four groups: (a) individuals who received the drug of interest (drug A) and exhibited dyslipidemia; (b) individuals who received the drug A, but did not exhibit dyslipidemia; (c) individuals who did not receive the drug A and exhibited dyslipidemia; and (d) individuals who did not receive the drug A and did not exhibit dyslipidemia. The reporting odds ratio (ROR) with 95% confidence interval (CI) and *Z* score was calculated as per formulae 1–3:

$ROR = \frac{\frac{a}{b}}{\frac{c}{d}}$…………………………………………………..…. 1

$95\% \mathrm{CI} = \exp\left\{ \log\left( ROR \right)\pm1.96\sqrt{\frac{1}{a}+\frac{1}{b}+\frac{1}{c}+\frac{1}{d}} \right\}$………….. 2

$Z \mathrm{score} = \frac{log(ROR)}{\sqrt{\frac{1}{a}+\frac{1}{b}+\frac{1}{c}+\frac{1}{d}}}$…………..…………..…………..……….. 3

where *a*, *b*, *c*, and *d* refer to the number of individuals in each group, and log refers to the natural logarithm.

Supplementary Data.xlsx: Table S3

**Supplementary Table S3** Overall confounding effects of concomitant drug (drug B) on olanzapine-induced dyslipidemia in the FDA Adverse Event Reporting System (FAERS) data.

Individuals who received olanzapine were divided into the following four groups: (a1) individuals who received the concomitant drug of interest (drug B) and exhibited dyslipidemia; (b1) individuals who received drug B, but did not exhibit dyslipidemia; (c1) individuals who did not receive drug B and exhibited dyslipidemia; and (d1) individuals who did not receive drug B and did not exhibit dyslipidemia. The reporting odds ratio (ROR) with 95% confidence interval (CI) and *Z* score for olanzapine-induced dyslipidemia was calculated as per formulae 4–6:

$ROR = \frac{\frac{a1}{b1}}{\frac{c1}{d1}}$…………………………………………………..…. 4

$95\% \mathrm{CI} = \exp\left\{ \log\left( ROR \right)\pm1.96\sqrt{\frac{1}{a1}+\frac{1}{b1}+\frac{1}{c1}+\frac{1}{d1}} \right\}$………….. 5

$Z \mathrm{score} = \frac{log(ROR)}{\sqrt{\frac{1}{a1}+\frac{1}{b1}+\frac{1}{c1}+\frac{1}{d1}}}$…………..…………..…………..……….. 6

where *a1*, *b1*, *c1*, and *d1* refer to the number of individuals in each group, and log refers to the natural logarithm.
